# To Use or Not to Use: Exploring Therapists’ Experiences with Pre-Treatment EMA-Based Personalized Feedback in the TheraNet Project

**DOI:** 10.1007/s10488-023-01333-3

**Published:** 2024-01-23

**Authors:** Mila Hall, Lisa M. Lappenbusch, Emily Wiegmann, Julian A. Rubel

**Affiliations:** 1 Department for Clinical Psychology and Psychotherapy (Adults), Osnabrück University, Osnabrück, Germany; 2 Department of Psychology, University of Giessen, Giessen, Germany

## Abstract

Background: Using idiographic network models in psychotherapy has been a growing area of interest. However, little is known about the perceived clinical utility of network models. The present study aims to explore therapists’ experiences with network model-based feedback within the context of the TheraNet Project. Methods: In total, 18 therapists who had received network-based feedback for at least 1 patient at least 2 months prior were invited to retrospective focus groups. The focus group questions related to how participation in the study influenced the therapeutic relationship, how the networks were used, and what might improve their clinical utility. The transcribed focus groups were analyzed descriptively using qualitative content analysis. Results: Most therapists mentioned using the feedback to support their existingtheir case concept, while fewer therapists discussed the feedback directly with the patients. Several barriers to using the feedback were discussed, as well as various suggestions for how to make it more clinically useful. Many therapists reported skepticism with regards to research in the outpatient training center in general, though they were also all pleasantly surprised by being involved, having their opinions heard, and showing a readiness to adapt research to their needs/abilities. Conclusions: This study highlights the gap between researchers’ and therapists’ perceptions about what useful feedback should look like. The TheraNet therapists’ interest in adapting the feedback and building more informative feedback systems signals a general openness to the implementation of clinically relevant research. We provide suggestions for future implementations of network-based feedback systems in the outpatient clinical training center setting.

**Supplementary Information:**

The online version contains supplementary material available at 10.1007/s10488-023-01333-3.

Psychotherapy is a generally effective treatment for several mental illnesses (Barkham et al., 2021). However, some patients still do not benefit from this kind of treatment (Hofmann et al., 2012), with some patients even experiencing a worsening in symptoms after therapy (Strauss et al., 2021). Psychotherapists are not always able to recognize and appropriately respond to these kinds of deteriorations (e.g., Hannan et al., 2005). For this reason, data-based feedback has been implemented in the clinical context, wherein psychotherapists can receive additional information about their patients (Barkham et al., 2021).

Feedback exists in several forms, with the most heavily researched form being routine outcome monitoring, or ROM (Lambert et al., 2003, 2018). ROM provides therapists with information about how their patients are developing throughout therapy in an effort to avoid negative developments in patient outcomes (Lambert et al., 2018; Lutz et al., 2022). In some studies, the feedback is provided to the patients as well (de Jong et al., 2021). However, the most typical form of data-based feedback appears to be provided to the therapist, who then decides if and when to share the information with the corresponding patient (de Jong et al., 2021).

According to several meta-analyses, this kind of ROM feedback can help improve patient outcomes (de Jong et al., 2021; Lambert et al., 2003; 2018; Rognstad et al., 2023). However, providing feedback in the form of ROM during therapy is not the only option. Several studies also include feedback at the beginning of psychotherapy, which may help further increase the positive effects of ROM on patient outcomes (Cohen & DeRubeis, 2018; Deisenhofer et al., 2018; Lutz et al., 2019; van Bronswijk et al., 2021).

## Pre-treatment Feedback Options

***Personalized Advantage Index (PAI).*** One example of a pre-treatment feedback tool is the Personalized Advantage Index (PAI; DeRubeis et al., 2014), which uses large amount of data from previously treated patients to form predictions about what kinds of treatment would benefit that individual the most, and what their symptom development could look like throughout therapy (Cohen & DeRubeis, 2018; van Bronswijk et al., 2021). In combination with ROM, using a tool like the PAI at the beginning of treatment and following its suggestions can help improve patient outcomes, for example, by contributing to larger decreases in symptom severity (Deisenhofer et al., 2018; van Bronswijk et al., 2021).

***Ecological Momentary Assessment (EMA).*** Another helpful method for obtaining pre-treatment information about patients or future patients is Ecological Momentary Assessment (EMA). EMA involves people filling out questionnaires in day-to-day life multiple times and typically over a longer period of time (Trull & Ebner-Priemer, 2009). The design of these methods (i.e., how many days and how many times per day a questionnaire is filled out, what the questionnaire content is), varies greatly across studies and is largely dependent on the specific research questions being investigated (Hall et al., 2021).

EMA is particularly well-suited to measure everyday life because certain constructs vary greatly across time, including but not limited to affect and emotion regulation (O’Connell et al., 1998; Schuler et al., 2019; Solhan et al., 2009). EMA offers the opportunity to gain insights into how emotion regulation, for example, varies across time and in everyday life for a particular patient before getting to know them very well throughout therapy. This may be of particular interest in a clinical setting, since most diagnostic questionnaires focus of retrospective reporting of experiences. Retrospective recall of certain experiences may be prone to recall bias, where certain experiences or emotions are not remembered as accurately as others (Ebner-Priemer et al., 2006; Schuler et al., 2019; Stone et al., 1998). For this reason, EMA may be a promising addition to more traditional, retrospective questionnaires.

***Network models.*** Network models can be used to visually display statistical relations calculated fromEMA data. These relations can include correlations between different variables or symptoms. They are two-dimensional graphs which depict symptoms or other important characteristics as points in that two-dimensional space. These points are sometimes referred to as nodes (Borsboom, 2017). The relationship between two symptoms/characteristics is visually represented as a line connecting them, an edge. This edge typically varies in thickness and in color to represent the strength and valence of the relationship. Using network models to visualize patient data has been of increasing interest in the past years, with several researchers lauding it for its potential clinical applications (Lutz et al., 2019; Piccirillo et al., 2019; Robinaugh et al., 2016; Rubel et al., 2018).

***Network models as pre-treatment feedback.*** Using network models in clinical practice, as a form of feedback, has been a quickly expanding area of research. This aligns with findings from a study by Bastiaansen and colleagues (2020), in which one fourth of twelve clinical research groups surveyed explicitly chose network modeling as their preferred method of visualization for EMA data.

Several research groups focused on how network models may support case formulation efforts (Burger et al., 2019, 2021; Riese et al., 2021). Other studies focused more on reactions to this kind of feedback from different perspectives. For example, Hall and colleagues (2023) conducted a qualitative study of therapists’ reactions to patient feedback while first viewing and being trained to interpret associated network models. Other studies have incorporated both patients’ and therapists’ perspectives on the utility of this kind of feedback (Frumkin et al., 2021; Zimmermann et al., 2019). In cases where both perspectives were taken into account, therapists were typically more skeptical about the utility of the models for clinical practice (Frumkin et al., 2021; Zimmermann et al., 2019), in particular when asked if the models provided any additional or surprisingly new information (Frumkin et al., 2021). A study by Zimmermann and colleagues (2019) also found that response rates amongst therapists were very low, with only 15 of 1065 therapists contacted participating, and only 8 of those 15 therapists responding to the final questionnaire about clinical utility. Other projects explored potential uses of these EMA-based network models (among other visualizations) for in-session discussion with the patient, at the case level and within a more controlled environment, where use was codified into study protocol (Kroeze et al., 2017; von Klipstein et al., 2023). However, the specific investigation of therapist (and not study initiated/mandated) use of EMA-based network model feedback in a naturalistic setting has not previously been studied.

Though there is increasing interest in using this methodology as a tool in clinical practice, there are several challenges related to the validity of network models and inferences based on them. For example, a recent review of the network literature concluded that many papers using the network methodology often referred to “centrality measures” as a way to identify appropriate treatment targets (Contreras et al., 2019). Broadly speaking, centrality measures summarize how interconnected a specific node is within a network, though various different centrality measures exist and measure slightly different things (Bringmann et al., 2019). Whether these measures truly provide meaningful and helpful recommendations for structuring psychotherapeutic treatments is still unclear. For further reading on this topic, we refer readers to the following, but certainly non-exhaustive, list of literature: Bringmann & Eronen, 2018; Bringmann et al., 2019; Haslbeck et al., 2021; Lunansky et al., 2022; Rodebaugh et al., 2020; Spiller et al., 2020; von Klipstein et al., 2020.

## Effectiveness of Feedback

Despite the promise of EMA-based, pre-treatment network model feedbacks, very little research exists on its effectiveness. However, the research that does exist about feedback use in general points to one essential requirement to achieving positive effects: Using the feedback (de Jong et al., 2012; Rubel et al., 2017; Simon et al., 2012). In other words, studies looking to investigate whether or not network model feedback is beneficial must also investigate the extent to which that feedback was used by therapists.

Attitudes towards these kinds of novel methods play an important role in clinicians’ openness to using the resulting tools (Ellison, 2020; Piot et al., 2022; Soyster et al., 2023).

Several different factors may influence therapists’ choices to use or not use feedback. For example, Ellison (2020) found that clinicians were generally more open to using tools like EMA for more challenging cases. For less complicated cases, they preferred other, more traditional support systems such as supervision and cross-sectional diagnostic assessments (Ellison, 2020). Soyster and colleagues (2023) also found that clinicians with more confidence in their clinical skills were more skeptical of and reported more hurdles to using tools like EMA in practice. Piot and colleagues (2022), on the other hand, uncovered much more favorable clinician and researcher responses to using EMA in clinical practice. The main reservations regarding the use of EMA were related to how time-consuming or burdensome it could be, for both patients filling it out and clinicians interpreting it (Piot et al., 2022). EMA was also viewed with skepticism in two other studies (Frumkin et al., 2021; Zimmermann et al., 2019), primarily because clinical implications were perceived to be missing.

However, a recent study found that therapists’ initial reactions to EMA-based network feedback were multifaceted, with initial intuitive ideas for its uses focusing on case formulation and reacting positively to suggestions for discussing the models psychoeducatively with patients (Hall et al., 2023). In this study, therapists received a training session on how to interpret the models, followed by discussing potential clinical uses for the feedback. Riese and colleagues (2021), on the other hand, had a researcher join sessions with the therapist and patient as a way of outsourcing the burden of learning to interpret the complex network models. Overall, it seems clear that highlighting the clinical uses or implications and providing aides to help in the interpretation of the models is essential.

## The Present Study

The TheraNet Project (Hall & Rubel, 2022) is an ongoing mixed methods randomized control trial (RCT) conducted at an outpatient psychotherapy training center. Within TheraNet, therapists received EMA-based, pre-treatment feedback for patients randomized to the intervention group. The control group patients did not fill out EMA and their therapists therefore provided treatment as usual. More information about the EMA procedure and the study in general are published elsewhere (Hall et al., 2022).

**Fig. 1:**
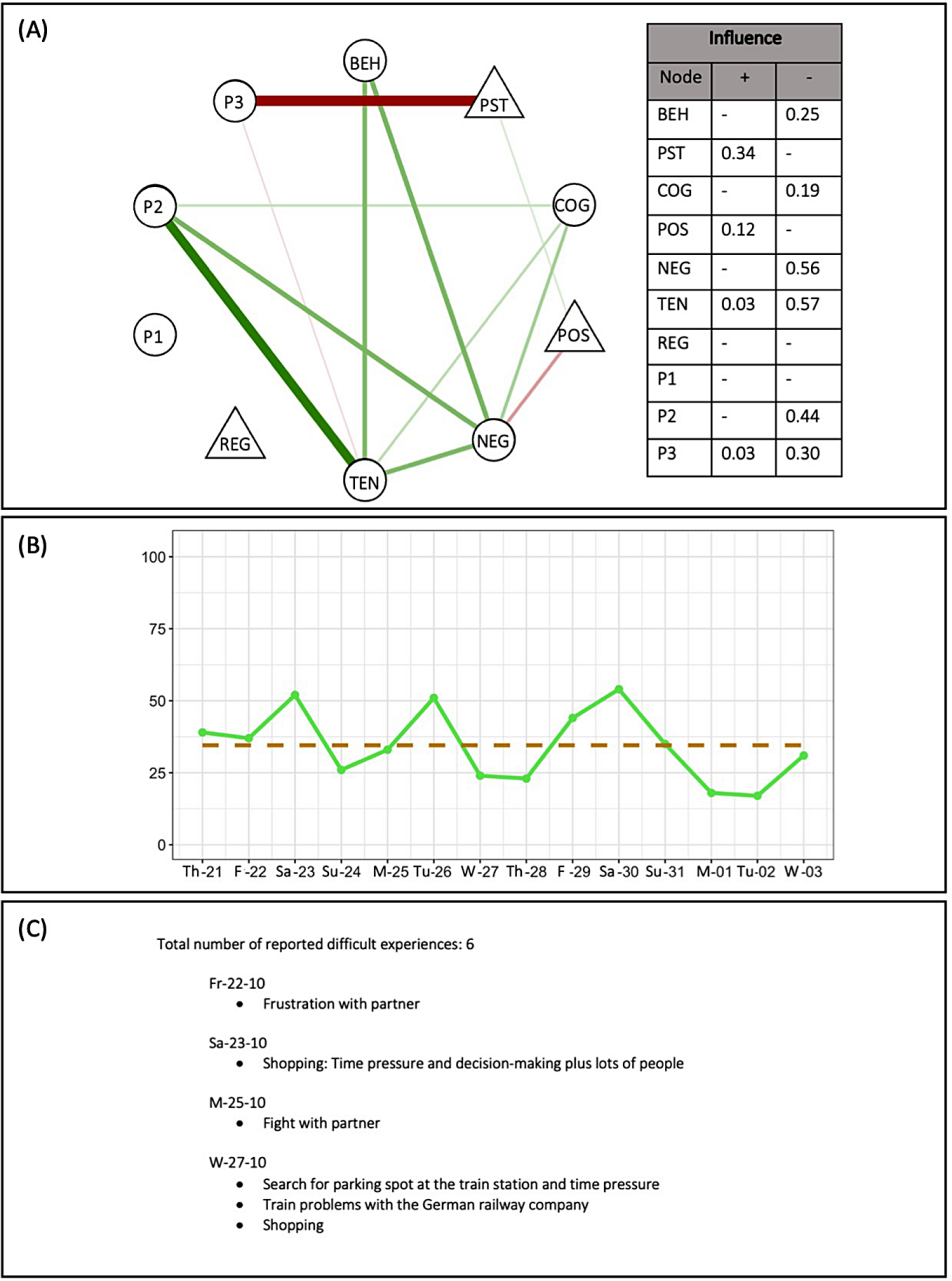
Example of a patient feedback, which is described in more detail in Hall et al., 2022. **(A)** Network: GraphicalVAR was used to calculate regularized partial correlation networks. Triangular nodes refer to positively phrased questions (resources), while circular ones refer to negatively phrased questions (potential problem areas). Green lines refer to positive partial correlations, while red lines refer to negative partial correlations. “Influence” is a network metric, similar to strength, that accounts for differently valenced questions (and is described in more detail in Hall et al., 2022). Item labels within the network refer to the following questions: “BEH” How much have you suffered from behaviors that you’d like to work on in therapy? “PST” To what extent have you engaged in activities that feel good to you? “COG” To what extent have you suffered from thoughts that you have not been able to turn off? “POS” How positive have you felt? “NEG” How negative have you felt? “TEN” How tense have you felt? “REG” How well do you feel you’ve been able to deal with your negative emotions? “P1-3” Personalized questions (always phrased negatively, phrasing chosen by participant; in this case “P1” To what extent did I pick at my face? “P2” To what extent did I feel overwhelmed by my fears? “P3” How listless was I?). Response scales are presented in Table 1. **(B)** Sleep diagram: Consists of the raw data points from the daily sleep quality EMA question (green points/line, date on the X-axis), where sleep was rated from “very poorly” (0) to “very well” (100) on the Y-axis. The average sleep quality reported for that patient within that timeframe is displayed via the brown dashed line. **(C)** Difficult Experiences: Reported chronologically and exactly as written by the patient, split up by day

The EMA-based pre-treatment feedback consisted of a network model that demonstrated the statistical associations between pre-treatment constructs, as well as a sleep diagram illustrating reported sleep quality and a list of stressful events that the participant endorsed during the pre-treatment period (see Fig. 1). The present study reports the results of retrospective focus groups conducted with therapists who had treated (or had started treating) at least one intervention and one control patient within TheraNet.

**Table 1:** Overview of EMA questionnaire. Abbr.: Abbreviation, as it appears in the translated feedback. More details on the EMA procedure, including the original German phrasing of the items, can be found in Hall et al., 2022 and its supplemental materials

Abbr.	Phrasing of Question *Always prefaced by “Since the last questionnaire…”*	Response Scale & Anchors
DIFF	Have you had a difficult experience?	Response scale: Yes/No If answered “Yes”, prompted with: “Briefly describe what happened…” Response: [open text box]
SLP	How well did you sleep last night?	Response scale: Sliding scale 0-100 Anchors: “Very poorly” | “Very well”
BEH	How much have you suffered from behaviors that you’d like to work on in therapy?	Response scale: Sliding scale 0-100 Anchors: “Not at all” | “A lot”
PST	To what extent have you engaged in activities that feel good to you?	Response scale: Sliding scale 0-100 Anchors: “Not at all” | “A lot”
COG	To what extent have you suffered from thoughts that you have not been able to turn off?	Response scale: Sliding scale 0-100 Anchors: “Not at all” | “A lot”
POS	How positive have you felt?	Response scale: Sliding scale 0-100 Anchors: “Not at all” | “A lot”
NEG	How negative have you felt?	Response scale: Sliding scale 0-100 Anchors: “Not at all” | “A lot”
TEN	How tense have you felt?	Response scale: Sliding scale 0-100 Anchors: “Not at all” | “A lot”
REG	How well do you feel you’ve been able to deal with your negative emotions?	Response scale: Sliding scale 0-100 Anchors: “Very poorly” | “Very well”
P1-P3	Personalized questions (always phrased negatively, phrasing chosen by participant)	Response scale: Sliding scale 0-100 Anchors: “Not at all” | “A lot”

The focus groups responded to several questions, including (1) how therapists believed their patients’ participation influenced the therapeutic relationship, if at all, (2) if they used the networks, and (3) how they thought the networks could be made more clinically useful. In this study, we aim to explore the actual use of the networks, any impact study participation had on the therapeutic relationship, what hindered therapists in using the networks, and what they thought could make the networks more clinically useful. These results will provide essential context information to future quantitative comparisons in TheraNet patients’ outcomes, once all ongoing therapies have ended. Additionally, these results provide first therapist-as-user experience information for future studies interested in investigating the clinical utility of network models.

## Method

### Setting

The TheraNet Project (Hall & Rubel, 2022) was implemented at an outpatient clinical training center in central Germany. Therapists working in this setting were in training and working through the hours required for licensure as a cognitive-behavior psychotherapist in Germany. Since TheraNet therapists were still in training, they were required to present and discuss each case with a licensed supervisor at least every fourth therapy session. The project was approved by the ethics commission of the Department of Psychology at the University of Giessen.

### TheraNet Procedure

Within the TheraNet Project, both therapists and their patients were recruited to participate in the study. Patients were randomized to either a control group or an intervention group. Feedback was only produced for the intervention group, since only they filled out the partially personalized EMA questionnaires, which were filled out for two weeks four times per day (see Table 1 for overview of EMA questionnaire). The EMA was initiated during the so-called probationary period, which precedes therapy sessions in the German healthcare system. The purpose of these sessions is to collect information required for the insurance claim. Between the probationary period and the beginning of therapy is a (typically) two-week long waiting period during which the insurance company reviews the claim before greenlighting the therapy.

The EMA-based feedback consisted of three sections: a contemporaneous network model that visualized the partial correlations between pre-treatment constructs, a sleep diagram showing self-reported sleep quality, and a list of reported difficult events reported during the pre-treatment period (see Fig. 1). An open-source software package, graphicalVAR (Epskamp, 2018), was used to construct a partial correlation network demonstrating the associations between pre-treatment constructs over time. For more details about the statistical analysis involved in the construction of the networks, we refer readers to Hall et al., 2022.

If a therapist had never received this feedback for any of their patients, they were invited to a one-time 1-on-1 training, where they were shown their first participating patient’s feedback and taught how to interpret it. These trainings were done on a rolling basis, with appointments being made individually at the earliest convenience for both the trainer-researcher and the therapist. Trainings were only conducted once per therapist. Once trained based on one patient’s feedback, therapists did not receive an additional training. For example, if an already-trained therapist took on a new patient, who was randomized to the EMA condition of TheraNet, they did not have to be trained again and instead received a printout of that new patient’s feedback once the EMA data collection was concluded. Further information about these trainings, including the training guidelines used and therapists’ first reactions to the feedback during their 1-on-1 trainings, can be found in Hall et al., 2023.

At least two months after therapists had received this 1-on-1 training (meaning they had received feedback for at least one patient), they qualified to participate in retrospective focus groups. The focus groups took place between October 2022 and January 2023. The groups began with an introduction to the topics to be discussed, as well as the interviewer transparently disclosing that the purpose of the discussion included and would benefit greatly from criticism and honesty (even if it was perceived as harsh). Next, the discussion questions were asked in order and as presented in Table 2 (for original German phrasing see Bilingual Table 2 in the Supplemental Materials). Therapists provided consent to have the groups audio- and video-recorded. These recordings were subsequently used to create detailed transcripts for each focus group.

**Table 2:** Focus group discussion questions

1. How did participation in the study affect the therapeutic relationship? (If there were difficulties with specific patients, which ones and why?)
2. Did you use the networks, and if so, how and with which patients?
3. How would you recommend altering the networks to make them more clinically useful?

Therapists were compensated separately for participating in different steps of the project: (A) for consenting to participating in general, research assistants would complete the manual entry of their patients’ diagnostic/research questionnaires; (B) for participating in a one-time 1-on-1 training, they received one free 1-on-1 supervision hour worth €~100; (C) for participating in the retrospective focus groups, therapists receive another free 1-on-1 supervision hour worth €~100.

More details about the EMA procedure and the TheraNet study protocol in general are described elsewhere (Hall et al., 2022; https://psyarxiv.com/8deyj/?).

### Sample Description

Overall, 34 therapists were invited via email to in-person focus groups. Focus groups were planned based on the collective availability of therapists who responded, with a final total sample of 18 therapists. Reason for attrition of therapists were scheduling conflicts (in 6 cases) and non-response to the original and follow-up emails (in the remaining 10 cases). The time between receiving the first feedback and the focus group was, on mean average 268 days (range = 84–386 days). Time between the first round of invitations and the focus groups ranged between 58 and 144 days, with a mean average of 88 days. First rounds of invitations were sent out in August 2022, and a final round (including therapists trained later and those who did not participate in previous focus groups) was sent out in December 2022.

In total, 5 focus groups were audio- and video-recorded, transcribed, coded, and discussed in consensus meetings. The focus groups took place October 2022 and January 2023. Each group included 3 to 4 therapists each, with an overall total of 18 therapists across all 5 groups. The sample consisted primarily of female therapists (*N*_female_ = 13, *N*_male_ = 5). Due to organizational reasons, not all therapists who were trained (*N*_*trained*_ = 24) were able to participate in focus groups. The focus groups therefore only included 75% of the full sample of TheraNet-trained therapists. The focus groups lasted between 45 and 70 min. Therapists had received feedback for anywhere between 1 and 6 individual patients (*M*_*mean*_ = 2.22, *SD* = 1.11).

### Qualitative Analysis of the Focus Groups

Focus groups were selected in order to encourage discussion among participants, andwere organized in two waves: the first three groups took place mid-October 2022 and the last two groups took place in mid-December and beginning of January. This wave structure allowed for more therapists to be included (i.e., because they met inclusion criteria later or because the suggested timeslots did not suit their schedules), but was also used as a way to assess the stability of the codebook.

The transcripts of the focus groups were analyzed using a combination of qualitative methodologies: qualitative content analysis according to Mayring (2010) and Consensual Qualitative Research by Hill (Hill et al., 2005). Qualitative content analysis systematically examines verbally communicated ideas and experiences and is well suited for exploratory questions such as those in this study (Mayring, 2010). The tenant from CQR that was used in the present analysis is that of discussing the codes and themes until consensus is reached amongst coders (Hill et al., 2005). The goal of the analysis was to inductively summarize and thematically group what therapists said, meaning that the categories were formed based on the text material and were not predetermined (Mayring, 2010).

Statements from the focus groups were grouped into meaningful sections, so called “chunks”, and were each assigned descriptive categories, or “codes”, in an iterative process documented with MAXQDA (VERBI Software, 2021). The coding process began after the first wave of focus groups. The resulting codebook was then used and updated as necessary for the second wave of focus groups. Though changes to the codebook between the waves were not systematically recorded, changes were minimal, with only a few new codes being added during the second wave. Once all the transcripts were coded and a parsimonious code structure had been created, the codes were clustered thematically in order to help organize the findings. All focus groups were coded independently by two raters, who then met and discussed until consensus was reached. These consensus meetings were supervised by the first author of this paper.

## Results

### Use of TheraNet Feedback

All therapists reported at least looking at the feedback. We refer readers to Table 3 for the exact frequencies with which these codes were used. In order to better understand use and what it looked like, we distinguished between direct, indirect, planned, failed, and less use of the feedback.

**Table 3:** Summary of how frequently codes were mentioned. Total frequency refers to the total number of times a code was used. Therapist frequency refers to the number of therapists mentioned that code (out of 18). Group frequency refers to the number of groups in which the code was mentioned (out of 5)

Section	Theme	Codes	Total freq.	Ther. freq.	Group freq.
Use of TheraNet feedback	Direct use	Feedback discussed with patient	17	9	4
		Patient asks for results	2	1	1
	Indirect use	Feedback wasn’t discussed with patient	6	5	4
		Feedback used to improve understanding of patient	15	10	4
		Findings corresponded to diagnostics/previous conversations	18	10	4
		Correlations seen as self-explanatory	2	2	2
		Justification of therapeutic interventions through network	7	3	3
		Feedback incongruent with clinical impression	2	2	2
		Unexpected correlation in network	3	2	2
		Connection sleep/difficult experiences unexpected	1	1	1
		Difficult experiences (text entry) most clinically useful	1	1	1
		Feedback enables better insight into everyday life	2	2	2
	Planned use	Therapist planned to discuss feedback with the patient	4	1	1
		Idea to use for insurance claim	2	2	2
		Idea to use to co-construct disorder model	2	1	1
	Failed use	Therapist tried to use feedback	2	2	2
	Less use	Therapist used feedback less over the course of study	4	3	1
Impact on therapeutic relationship	No impact on therapeutic relationship	Impact on therapeutic relationship unclear	5	5	4
		No impact on therapeutic relationship (i.e., because not discussed with patient)	9	9	5
		Prioritizing building the therapeutic relationship over feedback use	3	3	2
		Setting session agenda left up to patient	8	4	3
Hinderances to using TheraNet Feedback	Timing	Obstacle: Tight schedule in short-term therapy	1	1	1
		Obstacle: Still at the beginning of therapy	4	4	4
		Lost relevance due to delay	2	1	1
	Therapist expectations	Obstacle: Little benefit expected from discussion of feedback	1	1	1
		Therapeutic implications are missing from feedback	1	1	1
	Flaws in study design	Obstacle: Blind spot in patients‘ personalization	1	1	1
		Meaning of personalized items unclear (for patient and therapist)	3	3	2
		Reason for selection of items unclear	1	1	1
		Obstacle: Differentiation between correlation vs. clinical interpretation difficult	3	3	2
		Obstacle: Influence strengths too complex for therapists	3	3	3
		Obstacle: Interpretation made difficult by colors	1	1	1
		Obstacle: Interpretation not clear anymore	8	4	2
	Complexity	Obstacle: Feedback too complex for patient in-session	2	1	1
		Obstacle: Feedback too complex/time-consuming to explain	2	2	2
		Obstacle: Feedback too underwhelming for patient	1	1	1
		Obstacle: Feedback too general	4	2	1
Changes that could improve clinical utility	Format-level alterations	Idea to label personalized items	1	1	1
		Criticism for possibly judging “negative emotions” as negative	1	1	1
		Idea to rephrase “negative emotions” item	1	1	1
		Simpler disorder models easier to use	3	1	1
		Idea to have basic vs. advanced complexity levels of the networks	2	2	2
		Idea to separately depict positive and negative correlations	5	4	1
		Worries about more complexity through changes to the feedback	2	2	1
	Changes to EMA timing	Interest in seeing network changes (i.e. through pre-post comparisons)	6	5	5
		Idea for personalized EMA time-point/time-frame	3	3	3
	More personalization	Idea to have standardized items specified by patient	3	3	2
		Patient/therapist item personalization through conversations	7	4	3
		Patient/therapist item personalization through symptom lists	5	3	2
		Weight personalization overlaps between patient/therapist more	3	2	2
		Request to make EMA questionnaire more disorder specific	1	1	1
	More interpretation aides	Idea for more collegial exchange about feedback use	2	2	2
		Idea for more network interpretation aides	6	4	2

***Direct use.*** Direct use refers to therapists discussing the feedback directly with their patients in-session, which was reported by half of the therapists. This may have occurred because the therapists deemed the feedback as discussion-worthy independently, or (in the case of one therapist) because a patient asked directly whether their study results had been received yet.

***Indirect use.*** Indirect use was more multifaceted and difficult to operationalize, but generally referred to therapists looking at and using the feedback for their own purposes outside of therapy sessions. In these cases, therapists did not discuss the feedback with the patient directly (as stated explicitly by five therapists). Examples for indirect use were named within the code “feedback used to improve understanding of patient”, wherein therapists observed the feedback and used it as an information source more generally speaking. Ten therapists reported using the feedback in this indirect way. Ten therapists also reported a certain amount of overlap between information from the feedback and their clinical impression (either through diagnostic tools or their impression in previous therapy sessions), or that the relationships in the networks seemed self-explanatory or were generally compatible with existing models of psychological disorders (reported by two therapists).

Three therapists also discussed how they based parts of their therapy plan or particular interventions on information from the networks, for example:

*But I thought it was cool that her ability to regulate her emotions in particular had a super important influence on all kinds of variables [in the network], which made it possible to justify once again that this is an important factor that we should work on.*

(Group D, Therapist 12)

Two other therapists, on the other hand, reported exactly the opposite in the code “Feedback incongruent with clinical impression”. Similarly, two therapists reported this mismatch more specifically within the network, that a specific correlation appeared that they did not expect. Another therapist referred to an unexpected connection between sleep quality and difficult experiences, which also influenced how or whether they discussed it in-session:

*So I guess she had difficult experiences and then she slept better than usual. That didn’t fit somehow, or at least it didn’t make sense logically. And that’s why I, then I didn’t go into it in detail with her.*

(Group B, Therapist 6)

One therapist also stated, that they found the difficult experiences component of the networks to be the most clinically useful part of the feedback. Two therapists also stated that the feedback in general helped them gain better insight into their patients’ everyday lives, outside of therapy.

***Planned use.*** In some cases, therapists stated that they intended to use the feedback in upcoming therapy sessions or that they would want to use the feedback to write their insurance claims. Whether or not the therapists used the feedback indirectly was hard for them to tell, but they showed a general interest in concrete uses of the feedback (i.e., to co-construct a model of patients’ psychopathology or for the insurance claim). There were several barriers that hindered these ideas from being implemented, which will be discussed later.

***Failed use.*** Lastly, two therapists also reported trying to use the feedback in their therapies, but ultimately said they were not able to do so successfully. In one case, the therapist attempted to explain the network to her patient, but he was not able to understand it. In the other, the therapist tried to discuss the selected personalized items, but the patient could no longer remember why he had selected them.

***Less use.*** One therapist reported using the feedback less over the course of the study. With the first patients, she reported using the feedback indirectly, for her own case concept, and discussed it with one of her patients in-session. However, with later patients, she recognized that she used it less, primarily because the interpretation of the network was no longer clear to her. Other factors that influenced therapists’ use of the feedback will be explained later.

### Impact on the Therapeutic Relationship

When it came to the influence of patients’ participation in TheraNet on the therapeutic relationship, therapists stated that there was either no impact or at least no noticeable change. The remaining therapists nodded along in agreement^1^ during the groups, but did not verbally agree (which explains the discrepancy in the frequencies shown in Table 3). However, since TheraNet did not require the use of the feedback in any specific way, the therapists’ decisions on how they used the feedback (i.e., indirectly vs. directly) may have influenced this sense of stability in the therapeutic relationship.

There were three cases in which therapists reported prioritizing building their relationship with the patient over specific interventions, including discussing the networks. In fact, four therapists attempted to achieve this by letting their patients decide what the session agendas would be:

*The patient has an adjustment disorder due to a conflict that was relatively specific and she came to each of our sessions with situations that were very stressful for her. So it was more important for me to explore those situations and to work on them with her. Not to specify the topics in such a theoretical way. [It is] possible, theoretically, that [if the feedback had been discussed] she could have felt like her therapy was no longer about the situations she wanted to talk about, but that topics in therapy were being imposed upon her externally. It was important to me that she could come with her issues and we would address them.*

(Group A, Therapist 3)

### Hinderances to Using TheraNet Feedback

***Timing.*** Most hinderances to using the TheraNet feedback were discussed as it related to its use in-session, with the patient. For one therapist, this included time-factors such as perceiving the short-term therapy (24 sessions) as too tight of a time window, given the amount of problems the patient wanted to work on:

*I had my treatment plan and […] knew that it would be a relatively short process for her, that is, we would get there in short-term [therapy]. And I think I was under pressure to somehow get through what I had to do in that time, because I had the expectation, and so did she, that we would get through it in 24 h. I think that was the main reason why I didn’t use an hour for [the feedback].*

(Group B, Therapist 4)

Four therapists reported not having used the feedback yet, because they were still at the beginning of therapy. This was most commonly used as an explanation for why the feedback had not been used yet, but where therapists planned to use it in-session. However, one therapist stated having wanted to use it at the beginning of therapy, having postponed it, until it lost its relevance. In this case, the therapist no longer planned to use the feedback in-session.

***Therapist expectations.*** Therapists also voiced certain expectations related to the feedback’s utility, which hindered its use in-session. For example, the code “Obstacle: Little benefit expected from discussion of feedback”:

*Maybe […] that it wasn’t clear enough in my mind, how would my patient benefit if I discussed this more intensively right now? So I think I probably didn’t expect enough benefit for that.*

(Group C, Therapist 10)

Another therapist more specifically discussed the fact that direct clinical implications were missing from the feedback, which hindered her in discussing it in-session.

***Flaws in study design.*** Therapists also commented on several flaws or areas for improvement within the study. For example, they commented on the personalization procedure used in TheraNet for the patients’ EMA questionnaires. In this procedure (described in more detail in [blinded for review]), patients had the option to choose up to three questions in areas that they perceive as relevant. One therapist remarked on the fact that patients may have a blind spot with regards to what is truly relevant:

*I think some patients would pick out [personalized items] well on their own and others would leave out relevant things corresponding to their own blind spots.*

(Group A, Therapist 1)

Additionally, three therapists commented on the fact that, in some cases, there was confusion about the personalized items when they attempted to discuss it with the patients. This occurred when patients no longer remembered why they chose the personalized items, or that the meaning of the personalized items was no longer clear to them. All of these aspects hindered therapists in their use of the network models, both indirectly and directly.

Therapists also commented on difficulties in the interpretation of the network. Three therapists found it difficult to differentiate positive/negative correlations from clinically beneficial/detrimental correlations in the networks. The metric “influence” ([blinded for review]) was developed to help in this differentiation, but these therapists reported it being too difficult to understand for routine use:

*[I would have wanted] it to be a bit more intuitive, that you didn’t have to look at the [influence] table on the side and then think okay, that was in that direction and then in that direction. And then… I just didn’t use that.*

(Group C, Therapist 9)

One therapist reported that the confusion in the interpretations may have come through the colors used in the network (green for positive correlations, red for negative correlations). Four others said that the interpretation was generally not clear to them anymore since their interpretation workshop.

In addition to general study design-related hinderances, therapists also commented on the level of complexity of the feedback as a barrier to using it, stating that the feedback was too complicated or too simple, depending on the patient. For example, two therapists presumed that the feedback would be too complex or too time-consuming to explain in-session and therefore did not attempt to use it directly. Another therapist stated that the feedback was too complex for her patient when she attempted to discuss it in-session:

*We then discussed [the network]. But I couldn’t go into that much depth. That’s when I noticed that it was too overwhelming for her.*

(Group E, Therapist 15)

However, other therapists also mentioned that they felt the feedback was too simplistic to discuss with their patient(s) or that, in one case, it would underwhelm them. These opposite reactions to the complexity level of the feedback were reported in relation to therapists’ perceptions of patient characteristics.

*If I had discussed such banalities with her, I think she would have thought, “she’s not challenging me here” […].*

(Group B, Therapist 6)

Two therapists’ perceptions of the feedback was also that it was too general. In this case, this perception was not tied to patient characteristics, but represented the perspective of the therapists themselves. The standardized questionnaire, in particular, appeared several times in these comments:

*So I found [the network] to be very generic actually: when I’m super tense, I don’t feel so good. If I can regulate well, I have more positive affect. That was the central statement here and […] that’s just, yes, that’s how it is.*

(Group A, Therapist 1)

### What would have made the TheraNet Feedback more Clinically Useful?

***Format-level alterations.*** Upon being asked how they would alter the feedback to make it more useful, therapists had many different suggestions. Some of them related to the formatting of the feedback and what it looked like visually. For example, one therapist reported wanting the personalized items to have personalized labels. In its current format, personalized items were labeled P1, P2, and P3. A legend underneath the network contained the specific item chosen by the patient.

Another therapist voiced concern about the phrasing of the standardized question about negative emotions, stating that it could be construed as meaning that emotions that are unpleasant are “bad”. He therefore suggested rephrasing the question to avoid this misunderstanding.

Several comments related to the complexity of the feedback. One therapist repeatedly mentioned the ease of use of simpler models, particularly during psychoeducation. As mentioned previously, therapists differed in how they perceived the level of complexity in the networks and how they expected their patients to react to it. As a response to these mixed perceptions of how different patients would respond to the current level of complexity, the idea to be able to select the level of complexity was mentioned:

*Maybe one possibility would be a model with different levels. Because complexity has the advantage that you can somehow transfer it into a simple model, which you can show to patients, and at the same time you have a complex model, which you have more for the therapist.*

(Group A, Therapist 3)

In one focus group, the idea of separately presenting positive and negative correlations in an effort to reduce complexity was discussed. However, this suggestion was controversial within the group and was also followed up with concerns about the complexity increasing through this suggested change.

***Changes to EMA timing.*** In every focus group, an interest was shared in seeing how networks potentially changed either during or after therapy. In order to do this, EMA would have been repeated in order to produce a new or updated network for the therapists to use. According to one of the therapists, knowing that another future EMA round was going to occur would have increased her motivation to discuss it concretely in-session. This interest seemed to be connected primarily to checking whether or not the therapy plan was truly changing the patients’ day-to-day lives, as a measure of treatment success:

*I think I would find it totally exciting to do the [EMA] assessment again later, another two weeks, I don’t know starting at session 24 or so. So somewhere in the middle of the therapy, depending on how long the therapy lasts. Because I think you would also see changes and then it would be interesting, of course.*

(Group E, Therapist 18)

Three therapists also mentioned wanting to have the option to postpone the initial EMA data collection in order to have time to prepare their patients for this kind of protocol. They voiced concerns about their patients potentially needing more instruction before being able to reliably and validly report on certain emotional phenomena. One therapist also mentioned an interest in being able to flexibly reduce the compliance requirement for the feedback so that patients who are not able to reach the required compliance rate due to having a lower level of functioning could also benefit from their therapists receiving feedback.

***More personalization.*** In addition to being able to alter the timing of the EMA, therapists also generally voiced an interest in being able to personalize the contents of the EMA questionnaire. For example, there was a suggestion to let patients specify more details about the standardized items, echoed by three therapists:

*Maybe at the beginning, when they also determine P1, 2, and 3 [the personalized questions], you could include behavior, their own [problematic behavior, a standardized question], in parentheses, for example. And when they fill in the questionnaire every day, it just says in the parenthesis what is meant by it.*

(Group B, Therapist 5)

Therapists also discussed changes to the personalization procedure used for the EMA questionnaires. The possibility of involving the therapists in the selection of the personalized items was discussed frequently. Though the exact procedure through which this would work best was debated, several ideas were mentioned: using symptom checklists to be filled out by both the patient and therapist, deciding which items were relevant through an in-person conversation, making items available based on the patients’ diagnosis (to make the questionnaire more disorder-specific). The idea to weight items differently depending on whether or not both the therapist and patient had selected the item was also mentioned by two therapists. Overall, it was clear that therapists wanted to have the option to be more involved in the selection of personalized items.

***More interpretation aides.*** Lastly, therapists requested more support in the interpretation of the network models, either through informal intervision or through easily accessible interpretation aides. Four therapists stated, that simple and clear interpretation aides would have helped them utilize the feedback in-session by refreshing their memory. The suggested interpretation aides included simple and easy-to-read posters in the therapist work-rooms, one to two sentences printed directly underneath the network models, and/or highlighting the most important correlations within the network visualization.

## Discussion

In the present study, therapists’ perceptions of anduse/non-use of EMA-based network model feedback within the TheraNet Project was explored. Therapists varied greatly in how they made use of the TheraNet feedback. Though half of the therapists reported using the feedback directly, in-session, with their patients, several others mentioned using it for their own understanding of the patient (i.e., as a way to support their case formulation). They did not report any noticeable changes to the therapeutic relationship as a result of their patients’ participation in the study. However, therapists also mentioned several hinderances to using the feedback in-session, related to the timing, their own expectations, the study’s design, and the feedback’s level of complexity. Many of the suggestions to make the feedback more clinically useful related to increasing the amount of personalization, and how much the therapists could influence the personalization. In general, therapists appear to be curious and open to using EMA-based network feedback, though this study makes clear how important it is to involve therapists in the design and implementation thereof, as important stakeholders in this kind of research.

### Clinical use of Networks in Previous Studies

As mentioned previously, there is some new and ongoing research related to the clinical utility of psychological network models, particularly those based on patients’ EMA data. At the timepoint this paper was written, the majority of these projects have begun to present examples from these projects in the form of case studies or proof-of-concept papers (Hall et al., 2022; Kroeze et al., 2017; Riese et al., 2021; Rubel et al., 2018; Scholten et al., 2022; von Klipstein et al., 2023). Studies by Lutz and colleagues (2015; 2018; 2019) include network models as part of a larger battery of information provided to therapists throughout therapy, but did not directly investigate the use of networks specifically.

Analysis of the training sessions from the TheraNet Project (Hall et al., 2023) showed that several themes were identified in the discussion of the potential uses of EMA-based feedback: case conceptualization, therapy planning, and psychoeducation. The themes identified in TheraNet trainings also mirror the suggested network uses in several of the studies mentioned in the previous paragraph. Kroeze and colleagues (2017) present network models as a tool for psychoeducation to be used in-session, with researchers attending these sessions as well, so as to provide accurate and reliable interpretations. This provides an example for how the difficulties with interpretation may be handled differently: by having a researcher take on the responsibility for interpreting the models instead of teaching therapists, as was done in the TheraNet Project.

In the case example presented by von Klipstein and colleagues (2020), the use of the aforementioned program (Kroeze et al., 2017) is presented in more detail. The EMA-based feedback, including a network model, were discussed in-session and as a means to strengthen case formulation (von Klipstein et al., 2020). In these two papers, the patient perspective is also more actively incorporated, which was not integrated into the TheraNet Project in a systematic way. In the previously mentioned case example, the patient was able to voice his perspective on the use of the EMA-based feedback, generally stating that he learned a lot from it (von Klipstein et al., 2020). However, he also highlighted having wanted more time to digest the findings, particularly of the network model. This statement aligns with the concerns of TheraNet therapists regarding the complexity of the network models. However, given that some therapists in the present study stated that the opposite could be true too (i.e., that the patient would feel underwhelmed by the network), this may point to a need to be able to flexibly adjust these models’ complexity levels, depending on who is viewing them.

One of the themes mentioned in the TheraNet training sessions (Hall et al., 2023) that has been explored less in other studies is therapy planning. Several papers suggest this as a potential clinical use for network model metrics, such as centrality measures (Robinaugh et al., 2016; Rodebaugh et al., 2018; Rubel et al., 2017). However, this specific use has not been explored concretely in a naturalistic setting, though simulations show that this might be a promising approach to planning therapy (i.e., prioritizing more central nodes; Robinaugh et al., 2016). Additionally, the complexity of these kinds of metrics should be carefully considered given that TheraNet therapists scarcely reported using the influence metric and generally wanted more interpretation supports.

When therapists discussed possible changes to the TheraNet procedure, they mentioned being involved in the selection of items alongside the patients, with the possibility of weighting items more if both therapist and patient chose it. Weighting items differently is an option, if Bayesian network modeling is used (e.g., PREMISE; Burger et al., 2021). Therapists also mentioned being interested in how their patients’ network models changed during or after therapy, which can be tested using permutation tests (van Borkulo et al., 2023). Future research may consider investigating the use of these different methodologies in clinical context, including but not limited to the therapist-as-user experience thereof.

In addition to alternatives to the statistical methodology used in TheraNet, options besides EMA may also be worth considering to create network models. For example, using a method named perceived causal relations (PCR; Frewen et al., 2012) allows participants to draw networks, depicting their subjective perceptions of how different things relate to one another. This PCR method has been adapted for clinical use by Klintwall and colleagues (2023), in order to include psychological variables like symptoms. This method no longer relies on EMA-like data, but instead focuses on the construction of so-called perceived causal networks (PECAN; Klintwall et al., 2023). Using a method like PECAN instead of EMA for future studies on personalized networks for clinical practice would potentially address some of the therapists’ comments regarding further item personalization being useful.

In their seminal research, Schiepek and colleagues (2015) use a method similar to PECAN to explore the dynamical systems of patients’ symptomatology and resources. This dynamical systems approach and related research have the primary goal of identifying self-reinforcing feedback loops within these ever-changing systems, that can contribute to maintaining a patient’s mental illness or that may represent helpful feedback loops (i.e., learning processes; Schiepek et al., 2015; Wittenborn et al., 2016). These models are then regularly checked and updated throughout therapy, in part using personalized EMA (Schiepek et al., 2022). The highly personalized approach taken by Schiepek and colleagues (2022) mirrors the wishes mentioned by the therapists in the present study, to be more involved in the personalization but also to be able to adapt more aspects of the EMA to the individual patient. For example, this additional level of personalization could be achieved by allowing the EMA questionnaire to be co-constructed by both the therapist and the patient.

### Flexibility: Benefits and Drawbacks of Naturalistic Studies

The flexibility of how and if therapists used the TheraNet feedback had benefits and clear drawbacks. The training workshops conducted at the beginning of the study were an attempt at standardizing therapist competencies when it came to their understanding of the feedback, particularly the networks (Hall et al., 2023). However, the training provided was extremely short and may not have provided enough opportunities for the therapists to practice the interpretations. In a systematic review by Henrich and colleagues (2023), instructor-led training and web-based self-guided training both showed favorable effects on therapists’ competences. However, given the short and one-time nature of the TheraNet Project’s training, it may not have had the same favorable effects as the training programs reviewed in the aforementioned review (Henrich et al., 2023). Additionally, Henrich and colleagues’ review showed how important additional supervision can be in building the competences involved in using a new technique. Though therapists were encouraged to contact the researchers if they had questions, none of them did so. The TheraNet therapists’ supervisors were not involved in the study, which may also have led to different prioritizations in the use of the feedback.

Providing additional guidance as to how and with which patients the feedback should be used (i.e., in supervision or with the help of clinical support tools) may have increased usage. In some cases, therapists chose to use the feedback when it matched their clinical impression. The fact that TheraNet therapists independently chose with which patients to use the feedback presents a new general dilemma: whether these intuitive decisions were “correct”. Some therapists reported feeling more torn about using the feedback when it was contradictory, while others used this as an impetus to discuss it with their patients directly. Therapists varied in how their clinical impression influenced their inclination to use the feedback, aligning with previous research about how therapist characteristics influence feedback use (Rubel et al., 2017). This amount of flexibility allowed therapists to autonomously decide what was important, though previous research shows that clinical intuition can be dubious and misleading (Walfish et al., 2012).

In the TheraNet Project, a naturalistic approach was taken to the clinical utility of EMA-based network model feedback with the priority being on flexibility and freedom for the therapists involved. Despite the fact that the therapists’ intuitive decision-making process about how, when, and with which patients to use the feedback might have been flawed, this was a way of prioritizing the researcher-clinician relationship. In a large-scale, government-issued study of psychotherapy trainees in Germany, Strauss and colleagues (2009) identified the lack of “having a voice” as a major point of criticism within training. This sentiment of clinicians wanting to be heard mirrors the discussion around the researcher-practitioner gap, wherein communication is often viewed as one-sided (i.e., researchers exclusively telling practitioners what to do; Mischel, 2008; Teachman et al., 2012).

More rigorously controlled or manualized psychotherapy studies are often touted as the gold standard in psychotherapy research. The TheraNet Project may represent one end of the spectrum with regards to how manualized the use of the feedback was (i.e., not at all), instead prioritizing the motivation and sense of autonomy of participating therapists. Interestingly, Eifert and colleagues (1997) suggest that perhaps manualization and personalization of therapies can coexist, for example by using manualized treatments in theory-driven, empirically-guided and personalized ways.

### Institutional and Systemic Factors

Trainee psychotherapists, such as those included in this sample, are also in a unique professional situation. Several studies show that trainees working/training in several different countries and healthcare face similar high levels of stress (Engel et al., 2015; Heinonen et al., 2022; Pakenham & Stafford-Brown, 2012; Richardson et al., 2020). Higher financial insecurity, for example, has been shown to be highly correlated with life stress amongst trainee therapists (Heinonen et al., 2022).

Regulations and requirements within psychotherapist training vary greatly depending on country-wide legal regulations and/or institutional rules. In Germany, where the TheraNet Project was conducted, limited research has systematically explored the stress levels of psychotherapist trainees. However, one larger survey of German psychotherapists in training found that, 77% of the 2500 trainees surveyed were satisfied with the atmosphere in their training institutes, though they included answers of “quite satisfied” in this calculation (Nübling et al., 2020). This satisfaction rating differed greatly from that found in a different online survey, where just under half of the therapist trainees reported being unsatisfied or very unsatisfied with their institutes (Hölzel, 2009). In yet another online survey, mostly institutional/system-level conditions were named as areas for improvement, such as wishes for stable work contracts with clear legal regulations, quality control at the institute and clinic levels, and improvement of financing opportunities (i.e., through loans or grants; Drüge & Schladitz, 2016). These areas for improvement may have contributed to the mixed satisfaction ratings discussed above.

In a qualitative study, Brewster and colleagues (2015) developed a process model for the implementation of innovative technologies in a healthcare setting. They specifically identified that improving the climate at the implementation site, for example by directly solving problems and adapting to the needs of workers, also improved how well these innovations were accepted (Brewster et al., 2015). Within the TheraNet Study, these problems included the financial duress under which many trainee therapists find themselves. In an effort to support therapists and reward them for participating in the study, compensation in the form of one-on-one supervision hours were provided. Perhaps if TheraNet therapists had seen specific and visible improvements from making use of the feedback, their opinions may have been more favorable overall, as Brewster and colleagues (2015) state this is an essential next step in the successful adoption of new technologies.

Psychotherapy trainees also represent a specific subset of healthcare providers with unique challenges given their dependence on their supervisors. The relationship between a trainee and their supervisor is very unique and greatly depends on individual characteristics of both (Reichelt & Skjerve, 2002; Wilson et al., 2016). Within the psychotherapy training context, supervisors are in a position of power to their trainees, due to technicalities such as the supervisor having the formal case responsibility, regardless of how collegial the supervision relationship is (Wilson et al., 2016). In this sense, not involving supervisors in the TheraNet Project (i.e., whether trainee therapists used the feedback) acknowledged this power dynamic by empowering the therapist to make decisions about use independently and without external pressures. However, trainee therapists benefit greatly from responsive supervisors (Axelsson et al., 2023; Rast et al., 2017). Future studies involving trainee therapists should bear this power dynamic in mind when designing studies related to the use of specific therapeutic tools, and may consider observing the role of supervisors in use/non-use.

Despite the particularities of trainee psychotherapists and the ecosystems within which they work and exist, we would hypothesize that similar results to those presented here would have been found in a sample of more experienced, licensed psychotherapists. Many of the points brought up by this sample of trainee therapists could also be mirrored by those in later stages of their careers. Though the obstacles described by trainee therapists may be specific to their training context, it appears that other obstacles such as increased caseload and stressful beliefs may replace these later on (Hellman & Morrison, 1987; Deutsch, 1984). In other words, the specific obstacles may change, but the fact that there are obstacles does not. For this reason, future research into the effectiveness and use of psychotherapy tools should aim to reduce the burden on clinicians as much as possible, while also providing specific and measurable benefits.

### Limitations & Future Directions

As mentioned in the previous sections, the level of flexibility and clinical decision making, and institutional/systemic factors should be considered in future research about EMA-based network model feedback. In general, more research is needed about the clinical utility of network models, particularly if empirically-based decision support tools for their use are to be provided in the future. Both patient and therapist perspectives, as well as their personal characteristics, may provide further information about what exactly informs use of this kind of feedback and its use. Furthermore, more evidence is needed with regards to whether network models help improve patient outcomes.

In addition to the need for further research on clinical utility and effectiveness, further research is also needed on the statistical validity of network modeling (i.e., difficulties with power, handling missing data, centrality measures, and statistical assumptions) (Bringmann & Eronen, 2018; Bringmann et al., 2019; Haslbeck et al., 2021; Lunansky et al., 2022; Rodebaugh et al., 2020; Spiller et al., 2020; von Klipstein et al., 2020). Statistical limitations such as multicollinearity, low stability, sparser networks being preferred by the selection model (which may remove clinically relevant correlations from the network) may account for the therapists reporting that the feedback was incongruent with their clinical impression. Given these difficulties related to the statistical modeling, it becomes difficult to accommodate requests for flexibly reducing the minimum compliance to the EMA procedure, as mentioned by one therapist in this sample. When compliance is lower and there are therefore more missing values, the difficulties related to the statistical robustness of models like networks automatically increase. Both the usability and statistics research on network models are essential, as they have the potential to inform and improve each other.

The TheraNet Project did not utilize a method to systematically check how the feedback was used for each patient. These detailed qualitative results from a small sample of TheraNet therapists provide informative first insights into this new field of research, but expanding upon and validating these findings with quantitative results will help provide evidence for the utility of network models in clinical practice. Additionally, the delay between therapists receiving their first feedback and participating in the focus groups varied greatly. Some therapists participated in focus groups approximately two and a half months after receiving their first feedback, while others had almost a year between their first feedback and the focus groups. This fact, in addition to the therapists’ own concerns related to not being able to remember how to interpret certain aspects of the networks, may speak to the need to provide more continuous training or more readily available interpretation aides, as opposed to a one-time training. Future research should explore whether there are differences in use based on what kind of training and/or interpretation aides are provided, as a way of identifying an ideal balance of added burden and added value.

Some therapists who were trained to use the feedback (as presented in Hall et al., 2023) also did not participate in the focus groups presented here. The reasons for this attrition are unclear, though anecdotally, this occurred due to non-response to recruitment emails, scheduling difficulties, or last minute cancellation due to illness. For that reason, among others, the present study can also not guarantee that qualitative saturation was reached. It is possible, that if more therapists had participated, the resulting code structure would look different. Future studies may choose to more systematically ask therapists at fixed time intervals about their experiences with feedback in order to avoid therapists forgetting how they used feedback or not remembering the details of a case as well.

Additionally, social desirability is most likely an important limitation in the present study. The focus groups were conducted by the first author of this paper, a fellow trainee psychotherapist who has a personal relationships with some of the therapists included in the sample. In order to mitigate the influence of these prior relationships and social desirability with all therapists, the focus groups were framed as a space within which to honestly and openly discuss use of the feedback as a tool to be improved. Nonetheless, it is still possible that social desirability played a role and that the therapists held views they did not feel comfortable sharing within the group. For this reason, future research should consider how therapists’ opinions may be captured in a more anonymous way.

Future research should consider implementing an easy-to-use system, wherein the use of this type of feedback can be captured more efficiently. For example, one way of doing so would be to prompt therapists to report feedback usage during the documentation of their therapy sessions. If implemented, this type of system should be easy to use and not place any significant extra burden on the therapists. Adding too many additional steps (i.e., by adding detailed paper-based documentation) should be avoided, and digitalized options that integrate into existing software solutions (i.e., simply adding a box to existing documentation software to check) should be prioritized or developed. By reducing burden and prioritizing the needs of therapists, they may be more likely to remain open towards staying involved in clinical science as they transition into later stages of their career.

## Conclusion

Therapists were able to use the TheraNet feedback in a variety of ways, both directly in-session and indirectly for their own understanding. This occurred within a flexible study, where the use of feedback was not mandated or rigorously controlled. Though these flexible conditions represent a methodological challenge, with therapists varying in how much and for which patients they used the feedback, they maintained an openness and curiosity towards integrating network models into clinical practice. Several barriers to using the feedback were discussed, including its user friendliness and difficulties with interpretations. These barriers may have also been influenced by other contextual factors related to their trainee status, which requires further empirical investigation. Future research looking to implement network models into clinical practice may use these results as the basis for the design and procedure of these studies. Our wishlist for future research in this area is as follows:

1. Prior to designing this kind of feedback study, consider what problems/difficulties therapists may face in their day-to-day work life. Try to focus on only one and how the feedback you would like to provide will actively help solve/alleviate it (both in the short- and long-term).
2. Consider running ease-of-interpretation experiments (for example, with a student population). Compare how quickly, accurately, and easily students are able to learn to interpret different kinds of network parameters/methodologies in a non-clinical setting. Pay particular attention to the use of causal language where it is not appropriate.
3. Be intentional about which network parameters, methodologies, centrality measures, etc. you will be using. Document how you will educate those who will receive the feedback about how to interpret it.
4. Involve stakeholders (i.e., therapists, patients, outpatient center leadership, supervisors) in the initial designing and tweaking of the feedback and EMA. Consider involving a user experience designer in this process.
5. Listen to and adapt the design before running a pilot study.

## Electronic Supplementary Material

Below is the link to the electronic supplementary material.

Supplementary Material 1

## Footnotes

1 At this stage, we would like to point out that we documented non-verbal behavior during the transcription process, such as nodding, consistently. It occurred most frequently during the question about the impact on the therapeutic relationship, probably in part because therapists did not say much on this topic in general. We did not mention it for other codes because it did not add further information beyond the things that were verbally communicated.
